# Human adipose tissue-derived stem cells alleviate radiation-induced xerostomia

**DOI:** 10.3892/ijmm.2014.1837

**Published:** 2014-07-08

**Authors:** XUEYAN XIONG, XIUJUAN SHI, FENGSHAN CHEN

**Affiliations:** 1 Laboratory of Oral Biomedical Science and Translational Medicine, Department of Orthodontics, School of Stomatology, Tongji University, Shanghai 200072, P.R. China; 2 School of Medicine, Tongji University, Shanghai 200092, P.R. China

**Keywords:** human adipose tissue-derived stem cells, stem cell therapy, regenerative medicine, hyposalivation, irradiation, transdifferentiation, paracrine

## Abstract

Hyposalivation is an intractable side-effect of radiotherapy for head and neck cancer. It is caused by the irreversible loss of acinar cells and decreased saliva secretion. However, this situation severely compromises the quality of life of affected patients. Currently, there is no effective treatment for this condition. In the present study, we developed a novel approach to regenerate the function of the irradiation-damaged salivary glands using human adipose tissue-derived stem cell (hADSC) intraglandular transplantation. ZsGreen-labeled hADSCs were adoptively transferred into Sprague-Dawley (SD) rat submandibular glands immediately following exposure to 18 Gy irradiation. A higher salivary flow rate (SFR) was observed in the hADSC-treated group. Tissue improvement, including angiogenesis, anti-apoptosis and anti-fibrosis, was detected in the hADSC-treated glands as compared to the untreated glands. Quantitative reverse transcription PCR (RT-qPCR) revealed a significantly higher expression of vascular endothelial growth factor (VEGF), hepatocyte growth factor (HGF), cyclooxygenase-2 (COX-2) and matrix metalloproteinase-2 (MMP-2) in the hADSC-treated rats. Furthermore, immunohistochemical analysis indicated that the hADSCs had differentiated into acinar and ductal cells in the rat submandibular glands. Thus, our results suggest that hADSCs are able to regenerate irradiation-damaged salivary glands through glandular transplantation.

## Introduction

Hyposalivation is affected by multiple factors and usually follows head and neck cancer therapy. Head and neck cancer is the fifth most common type of cancer, and represents approximately 6% of all cases and accounts for an estimated 650,000 new cancer diagnoses and 350,000 cancer-related deaths worldwide annually (1). Radiotherapy plays an important role in conventional therapy, either independently or combined with surgery, which results in a 5-year survival rate of approximately 50% for non-metastatic and locally advanced disease (2).

However, the salivary glands, which are frequently included in the radiation field, are severely damage by this approach. A large number of affected patients suffer from xerostomia, dysphagia, dental caries, fungal infection and malnutrition that consequently and severely compromise their quality of life. The irreversible salivary gland dysfunction has been attributed to a noticeable lose of acinar cells and the sterilization of primitive glandular stem cells (3). Artificial saliva substitutes and sialogogues represent the main clinical strategies used to relieve oral dryness; however, tehse strategies require frequent administration and are unpredictable in their efficacy (2,4). Therefore, studies have sought to regenerate irradiation-injured glands. Stem cell-based therapy is considered as a feasible approach to restore radiation-damaged salivary glands. Bone marrow-derived cells (BMDCs) may also hold promise for repairing the irradiation-damaged salivary gland (5,6). Nevertheless, there are unavoidable clinical issues associated with transplanting BMDCs, due in part to painful procurement, low yield rates and relatively low proliferation rates. Another source suggested as a candidate for the treatment of irradiation-induced hyposalivation includes cells derived from salivary glands. The function of irradiated mouse salivary glands can be rescued by an intra-glandular injection of primitive salivary gland stem cells (7). However, the self-renewal capacity of salivary gland stem cells is restricted *in vitro*, and as most patients treated for head and neck cancer are elderly, obtaining a sufficient amount of primitive salivary gland stem cells for clinical use is challenging.

Human adipose tissue-derived stem cells (hADSCs) are pluripotent stem cells obtained from human adipose tissue, and similar to BMDCs, they have the multipotential to differentiate toward osteogenic, adipogenic, myogenic and chondrogenic lineages (8). Moreover, hADSCs can be easily obtained from human adipose tissue or lipoaspirates, which cause little discomfort to the donor and have gained significant attention as a source of cell-based therapy. hADSCs have been investigated for the possible treatment of tissue defects and organ dysfunction in diabetes, pancreatitis, cardiac and neurological diseases (9–11). Furthermore, in a previous study, the potential toxicity and tumorigenicity of hADSCs was investigated in animals and humans and the hADSCs were found to be both non-toxic and non-tumorigenic (12). Based on the above study, we speculated that hADSC transplantation may be a promising strategy for stem cell-based treatment to alleviate xerostomia. This study aimed to determine whether hADSCs possess the ability to restore radiation-induced salivary gland dysfunction by local engraftment in a Sprague-Dawley (SD) rat model of irradiation-induced hyposalivation. The data presented in this study provide important new information for the potential application of hADSCs in the treatment of salivary gland dysfunction.

## Materials and methods

### hADSC ZsGreen labeling, flow cytometry and differentiation

The cells were seeded into T75 flasks and grown to 20% confluence. The medium was changed to Dulbecco’s modified Eagle’s medium (DMEM) with F12 (Gibco, Grand Island, NY, USA) that was supplemented with 10% FBS and the cells were incubated for 4 h. The purified lentiviral vector, ZsGreen, was added to the medium at a multiplicity of infection (MOI) of 20. The medium was changed to mesenchymal stem cell medium (MSCM; BioWit Technologies, Shenzhen, China) 8 h after infection. ZsGreen expression was examined by fluorescence microscopy 48 h after infection. The ratio of ZsGreen-positive cells in the infected hADSCs was determined using the captured images. Flow cytometry, as well as the adipogenic and chondrogenic differentiation of ADSCs were carried out as previously described (13). The cells were dissociated using 0.25% trypsin-EDTA and washed twice with PBS. The cells were then incubated with anti-human CD36 (eBioscience, Inc., San Diego, CA, USA), anti-human CD44 (eBioscience, Inc.) and corresponding isotype control antibody at 4°C for 30 min. Following incubation, PBS with 0.5% bovine serum albumin (BSA) was utilized to wash the cells 3 times. Flow cytometric analysis was performed using a FACSCalibur flow cytometer (FACSAria III; BD Biosciences, Franklin Lakes, NJ, USA), and the data were analyzed using FlowJo software (TreeStar Inc., Ashland, OR, USA). When the confluence of the cells reached 80%, the differentiation experiments were performed. For adipogenic differentiation, the medium was changed to adipogenic differentiation medium (DMEM/F12 supplemented with 10% FBS, 1 μM dexamethasone, 0.5 mM isobutyl-methylxanthine and 60 μM indomethacin). Adipogenic differentiation was examined by Oil Red O staining 14 days after induction. To induce chondrogenic differentiation, the cells grown in DMEM medium containing 500 ng/ml BMP-6, 10 ng/ml TGF-β3, 0.1 μM dexamethasone, 0.17 mM ascorbate-2 phosphate, 50 mg/ml ITS+ premix (BD Biosciences: 6.25 μg/ml insulin, 6.25 μg/ml transferrin, 6.25 ng/ml selenous acid, 1.25 mg/ml bovine serum albumin and 5.35 mg/ml linoleic acid). Chondrogenic differentiation was examined by Alcian blue staining 21 days after induction.

### Animals

Twelve-week-old male SD rats were purchased from Sino-British SIPPR/BK Laboratory Animal Co., Ltd. (Shanghai, China) and were used for the experiments. The rats were kept in a specific pathogen-free, micro-isolated environment at the Institute of Laboratory Animals, Tongji University, Shanghai, China, and allowed access to water and chow *ad libitum*. The rats were divided randomly into 3 groups as follows: group 1 (normal, n=30), group 2 (18 Gy irradiation, n=30), and group 3 (18 Gy + hADSCs, n=30). The Animal Research Committee of Tongji University approved the study and all the procedures that involved rats.

### Irradiation of the salivary gland

Prior to irradiation, all the rats were weighed and anesthetized using sodium pentobarbital (4.5 mg/100 g body weight). Subsequently, the rats in groups 2 and 3 were fixed on a special apparatus, with the head and neck exposed and other parts of the body protected. Single-dose irradiation at 18 Gy was administered using a 6-MV X-ray linear accelerator (2100C/D; Varian Medical Systems, Inc., Palo Alto, CA, USA) at a dose rate of 300 cGy/min at a focus to skin distance of 100 cm. Group 1 rats did not receive any irradiation.

### Transplantation of hADSCs

Immediately following irradiation, 1x10^6^ hADSCs in 0.1 ml PBS were adoptively transferred by subcutaneous injection into the submandibular glands of the rats in group 3 under anesthesia. Additionally, the same volume of PBS was injected into the glands of the group 2 control rats. Group 1 rats received anesthesia but no treatment or PBS.

### Saliva collection

One PE-10 tube (outer diameter, 1.00 mm; inner diameter, 0.65 mm) was inserted into the duct opening of the submandibular gland of the rats after receiving anesthesia. Salivary output was collected following stimulation with subcutaneous injections of pilocarpine (0.06 mg/100 g body weight) and placed in pre-weighed 0.5 ml microcentrifuge tubes. Saliva was collected for a period of 10 min, and the volume was estimated by weight, which assumed a specific gravity of 1.0 g/cm. The salivary flow rate (SFR) was assessed at 8, 16 and 24 weeks post-irradiation.

### Histological and morphological examination

At 24 weeks post-irradiation, the submandibular glands were harvested and washed for 30 sec in PBS and fixed with 4% paraformaldehyde (PFA) at 4°C overnight and embedded in paraffin. The paraffin-embedded glands were cut into 5-μm-thick sections and subjected to hematoxylin and eosin (H&E) or periodic acid-Schiff (PAS) staining. TdT-mediated dUTP nick end-labeling (TUNEL) staining (In Situ Cell Death Detection kit; Roche Diagnostic Corp., Indianapolis, IN, USA) was performed as previously described (14). The sections were ice-bathed in 0.1% Triton X-100 for permeation for 5 min and washed twice with PBS for 5 min. The sections were then incubated with a TUNEL reaction mixture for 60 min at 37°C in the dark. After the reaction, the sections were washed in PBS and stained with hematoxylin. The sections were then mounted with neutral gum before being examined under a light microscopy. Paraffin sections were stained by an immunohistochemical method using the blood vessel staining kit (Chemicon International, Inc., Billerica, MA, USA) following a previously published protocol (6). The sections were deparaffinized, rehydrated and rinsed with 10 mM sodium citrate (pH 6.0) solution 3 times in a 600-W microwave and cooled to room temperature. After blocking overnight at 4°C, the sections were incubated in 1:200 diluted rabbit anti-vWF polyclonal antibody at room temperature. After secondary antibody incubation and HRP reaction, the sections were examined under a light microscope. The percentage of surface occupied by blood vessels was counted under x400 magnification using 100 squares of 0.25 mm^2^ each. Five squares were scored from each gland, and subsequently the data were assessed using NIH ImageJ software (NIH, Bethesda, MD,USA).

### Quantitative reverse transcription PCR (RT-qPCR

Total RNA was extracted from the fresh enucleated salivary glands using TRIzol reagent (Invitrogen Life Technologies, Carlsbad, CA, USA), and cDNA was synthesized using the PrimeScript™ RT reagent kit (Takara Bio, Inc., Shiga, Japan). The relative expression levels of vascular endothelial growth factor (VEGF), hepatocyte growth factor (HGF), cyclooxygenase-2 (COX-2) and matrix metalloproteinase-2 (MMP-2) were determined using SYBR Premix Ex Taq (Takara Bio, Inc.) and an Applied Biosystems 7900HT device (Life Technologies, Inc., Grand Island, NY, USA). The primer sequences are presented in Table I. The average threshold cycles were determined from triplicate PCR cycle assays, and the relative expression levels were normalized to the endogenous control GAPDH.

### Immunohistochemical analysis

The freshly isolated glands were fixed in 4% PFA, embedded in optimal cutting temperature medium and sectioned at a thickness of 8 μm. The cryostat sections were air-dried at room temperature, post-fixed in 1% PFA and blocked in blocking buffer consisting of 5% normal goat serum (Gibco), 0.1% Tween-20, 3% BSA in PBS for 1 h at room temperature. The blocked sections were incubated with antibodies (diluted in blocking buffer) targeted against the following proteins overnight at 4°C: cytokeratin 7 (CK7, 1:250; Santa Cruz Biotechnology, Inc., Santa Cruz, CA, USA), cytokeratin 14 (CK14, 1:50; R&D Systems, Minneapolis, MN, USA), Na^+^-K^+^-Cl^−^ co-transporter type 1 (NKCC1, 1:250; Abcam, Cambridge, MA, USA) and α-smooth muscle actin (α-SMA, 1:50; R&D Systems). The slides were then washed in PBS prior to incubation with secondary antibodies that were conjugated to Cy-3. The slides were then washed in PBS and counterstained with 4′,6-diamidino-2-phenylindole (DAPI), and then mounted using 50% glycerol-PBS before being examined under a fluorescence microscope (IX71; Olympus Corp., Tokyo, Japan)..

### Statistical analysis

All data are expressed as the means ± SD. To determine statistical significance, one-way factorial analysis of variance (ANOVA) and Student-Newman-Keuls (SNK) analysis were conducted using SPSS version 17.0 software. A value of p<0.05 was considered to indicate a stastistically signficant difference.

## Results

### Characterization of hADSCs

**Figure 1:**
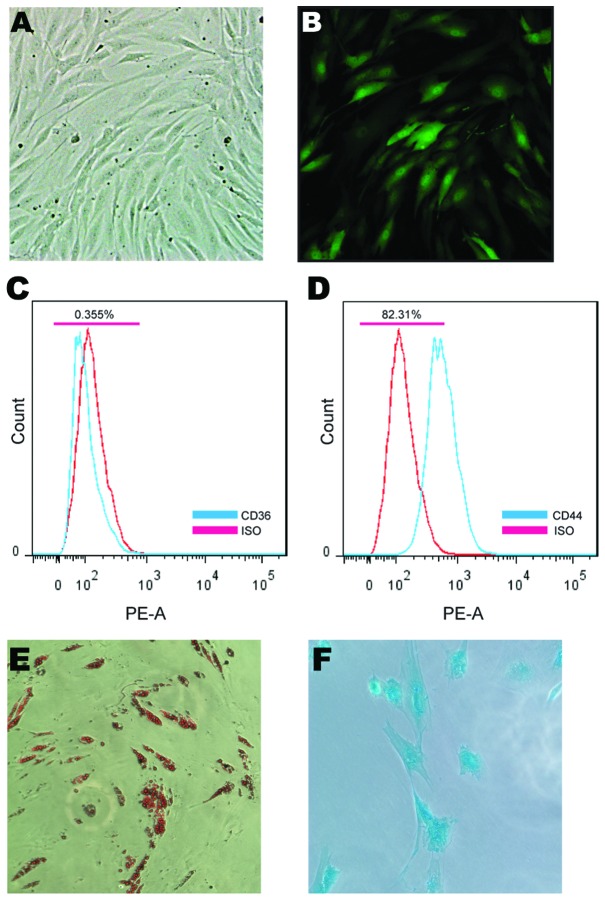
Human adipose tissue-derived stem cell (hADSC) ZsGreen labeling, flow cytometry and differentiation. hADSCs were transduced with ZsGreen by the suspension infection method. (A) At 48 h after infection, the infected hADSCs were in good condition, as shown by bright-field microscopy. (B) The rate of ZsGreen-positive cells (green signal) was approximately 80%, as shown by dark-field microscopy. Flow cytometric analysis of hADSCs for the expression of CD36 and CD44 was performed (blue). Cells stained with isotype control are also shown (red). hADSCs were negative for (C) CD36 and positive for (D) CD44. hADSCs had long, spindle-shaped, fibrocytic-like adherent growth. (E) At 2 weeks after induction, hADSCs in the adipogenic differentiation medium displayed accumulation of lipid droplets that were stained with Oil Red O. (F) hADSCs that were cultured for 3 weeks in chondrogenic differentiation medium stained positive for Alcian blue.

The morphology of the hADSCs [passage 3 (P3)] was fibroblast-like and the hADSCs were effectively infected with the lentivirus expressing ZsGreen (Fig. 1A and B). Flow cytometry was performed for the hADSC cell lineage classification prior to transplantation (Fig. 1C and D). Chondrogenic and adipogenic differentiation was also used to verify the multipotential differentiation capacity of the hADSCs used in this study (Fig. 1E and F).

### Functional improvement of hyposalivation by SFR detection

**Figure 2:**
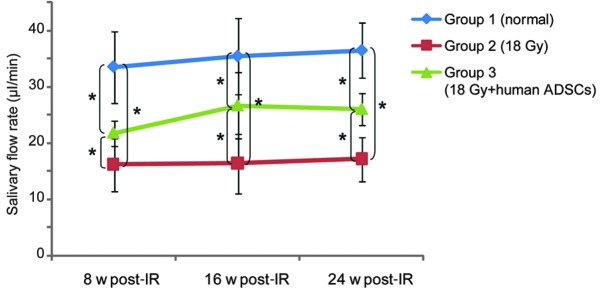
Salivary flow rate (SFR) at 8, 16 and 24 weeks post-irradiation. The SFR of the rats in group 3 was higher than that of the rats in group 2 (p<0.05), and recovered to 71.45% of the SFR of the rats in group 1 at 24 weeks post-irradiation. ^*^p<0.05. w, weeks; IR, irradiation.

To assay the functional improvement in radiation-induced hyposalivation, the SFR was measured at 8, 16 and 24 weeks post-irradiation. The SFR of the rats in group 2 sharply decreased by 48.27% of the SFR of the rats in group 1 at 8 weeks after radiography. Group 2 rats also showed persistent hyposalivation throughout the experiment, and maintained only approximately 50% of the SFR of the rats in group 1 at 24 weeks (p<0.05). At 8 weeks post irradiation, a decrease in SFR was also observed in the rats in group 3; however, this decrease was not as significant as that of the one of the rats in group 2 at 8 weeks after irradiation (p<0.05). In addition, the output for group 3 was 1.51-fold higher compared to that for group 2, and the SFR recovered to 71.45% of the SFR of the rats in group 1 at 24 weeks (p<0.05, Fig. 2).

### Injury tissue improvement and blood vessel regeneration following hADSC transplantation

**Figure 3:**
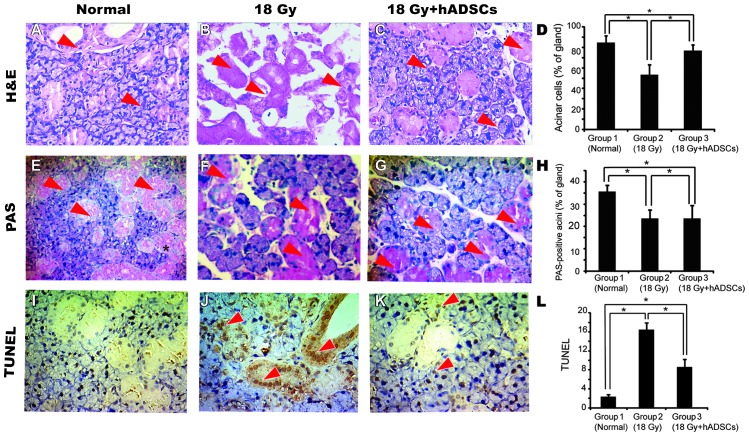
Injury tissue restoration by human adipose tissue-derived stem cells (hADSCs). (A-C) H&E staining: (A) Normal rat submandibular salivary gland showing mixed glandular tissue. Coagulative necrosis and interstitial edema were observed in the irradiated glands at 24 weeks. (B) The cytoplasm was homogeneously stained red, while the cell nuclei were atypical and split. (C) Cytoplasmic vacuoles and intercellular space dilation were observed in the hADSC-treated glands. Arrows indicate H&E-positive cells. (D) Mean percentage of acinar cells in the salivary gland. The paraffin section was stained with H&E and scored for acinar cells as described in the ‘Materials and methods’. Transplanted hADSCs increased acinar cells in irradiated submandibular glands. (E-G) PAS staining. PAS staining showing PAS-positive acini (E) in a normal gland, (F) an irradiated gland, and (G) a hADSC-treated gland observed 24 weeks post-irradiation. Arrows indicate PAS-positive acini. (H) The mean percentage of PAS-positive acini in the salivary gland. A greater number of PAS-positive acini was observed in the gland after hADSC transplantation as compared with the non-treated glands. (I–K) TUNEL staining. (I) The apoptotic cells were observed in a normal rat submandibular gland. (J) An increased number of apoptotic cells was observed in an irradiated gland by TUNEL staining, and involved both acinar and duct cells. (K) Apoptotic cells were observed to spread sporadically in the gland following hADSC engraftment. Arrows indicate TUNEL-positive cells. (L) Apoptotic cell scoring showed higher apoptotic activity in the irradiated glands than in the hADSC-treated glands. All scale bars, 50 μm; ^*^p<0.05.

Pronounced acinar loss occurred in the rats in group 2 by 24 weeks post-irradiation as compared to the rats in group 1. Extensive cellular and interstitial edema, cellular necrosis and blood vessel congestion were observed in the rats in group 2. Moreover, edema, blood vessel congestion, coagulative necrosis and fibrosis were observed in the irradiated glands. By contrast, the hADSC-treated salivary glands had a greater number of acinar cells that displayed a normal morphology. In addition, swelling of the parenchymal and mesenchymal cells, congestion and duct dilation were observed in the rats in group 3. No inflammatory cells were detected in group 3 by H&E staining (Fig. 3A–C). Additionally, PAS staining revealed a greater number of PAS-positive acini in group 3 compared to group 2 (p<0.05, Fig. 3E–G). An increased number of apoptotic cells was observed in group 2 based on the observations derived from TUNEL assay, which involved acinar and duct cells, while a sporadic pattern of apoptotic cells was observed in group 3 (p<0.05, Fig. 3I–K).

**Figure 4:**
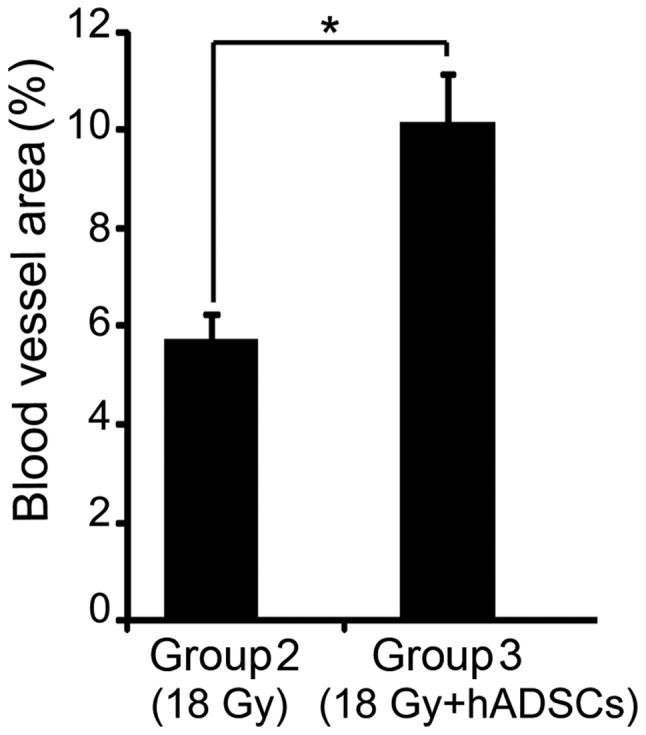
The area percentage assay of blood vessels. The area percentage of blood vessels in group 3 (18 Gy + hADSCs) was 1.59-fold higher than that of the vessels in group 2 (18 Gy) 24 weeks post-irradiation. ^*^p<0.05.

The frequency of the surface area occupied by the blood vessels in each gland was calculated by blood vessel staining. The area percentage in the hADSC-treated glands was 1.59-fold higher than that of the non-treated glands (p<0.05, Fig. 4).

### Paracrine effect of hADSCs

**Figure 5:**
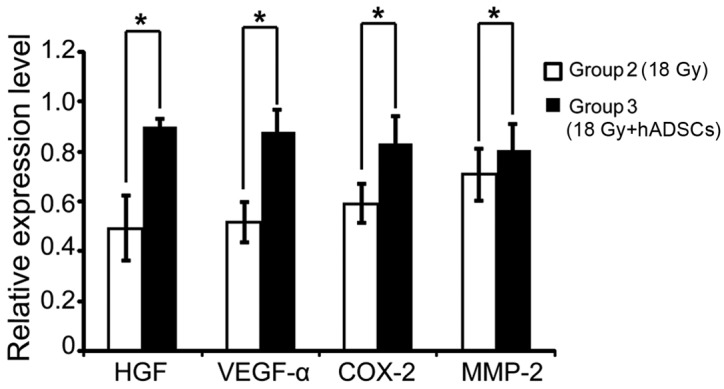
Factor contribution to injury tissue improvement. RT-qPCR indicated that the mRNA expression of vascular endothelial growth factor (VEGF), hepatocyte growth factor (HGF), cyclooxygenase-2 (COX-2) and matrix metalloproteinase-2 (MMP-2) was markedly increased in the glands from the rats in group 3 as compared to those of the rats in group 2 (p<0.05). ^*^p<0.05.

hADSCs secrete multiple cytokines and growth factors that may contribute to the functional reconstruction of injured organs (15). At 24 weeks following hADSC engraftment, total RNA was extracted from the freshly enucleated salivary glands, and RT-qPCR indicated that the mRNA levels of VEGF, HGF and COX-2 increased significantly in the rats in group 3 as compared to the rats in group 2. Moreover, MMP-2 expression showed no significant increment in expression (Fig. 5).

### hADSC differentiation into acinar and duct cells in the submandibular glands

**Figure 6:**
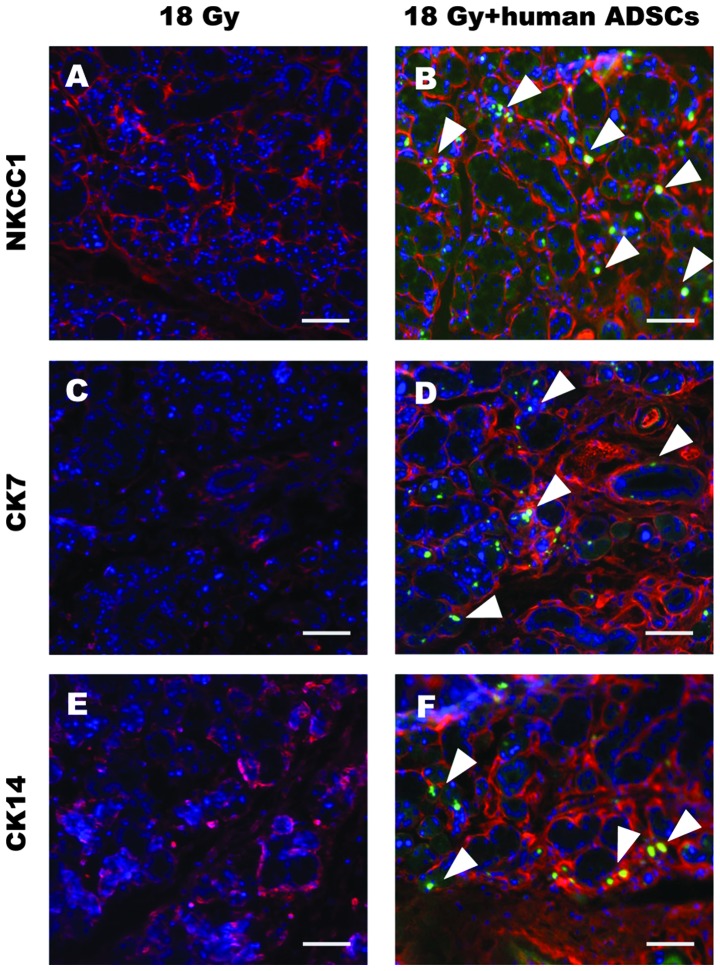
Double immunohistochemical staining in the salivary glands. All the nuclei were stained blue using 4′,6-diamidino-2-phenylindole (DAPI). Different salivary gland epithelial cell markers displayed red signals. (A and B) Na^+^-K^+^-Cl^−^ co-transporter type 1 (NKCC1): NKCC1 is the red signal surrounding the acinar surface. (B) Some of the NKCC1-positive cells were ZsGreen-positive in the hADSC-transplanted glands. (C) Cytokeratin 7 (CK7): No ZsGreen-labeled cells were found in the irradiated gland. (D) The ZsGreen-labeled cells were also found to express CK7, which is a ductal cell marker. (E and F) Cytokeratin 14 (CK14): Ductal cells were to express CK14, as shown by the red signal. (F) ZsGreen and CK14 double-positive cells were observed in the glands from the rats in group 3. Arrows indicate ZsGreen-stained cells; Scale bar, 100 μm.

Markers of the 3 main parenchymal cells in the salivary gland, including acinar, ductal and myoepithelial cells, were assayed by double immunostaining. At 24 weeks post-transplantation, ZsGreen-positive cells could still be observed in the submandibular glands from the rats in group 3. Moreover, ZsGreen-positive cells were located in cells that expressed NKCC1 (an acinar cell marker), CK7 and CK14 (duct cell markers), but they did not express the myoepithelial cell marker, α-SMA, Fig. 6).

## Discussion

Xerostomia remains a major challenge and complication of radiotherapy for head and neck cancer. In this study, we demonstrate the potential of hADCSs for rescuing radiation-damaged rat submandibular glands by intra-glandular transplantation. Our findings showed that engrafted hADSCs survived in the submandibular glands, and played a role in the protection against irradiation-induced cellular apoptosis, and promoted angiogenic activity. Moreover, a small proportion of hADSCs that expressed salivary gland cell makers was also detected.

Mesenchymal stem cells (MSCs) are found in a variety of tissues, including BMDCs (16), umbilical cord blood placenta (17) and fat (18). MSCs derived from human adipose tissue are referred to as hADSCs in this study, which present an accessible and available source for stem cell therapy, and have been applied in numerous animal and clinical trials (9). The application of MSCs for tissue repair can be systematically or site-directed. It has been reported that systemically infused MSCs localize within injured, inflamed and cancerous tissues. However, their efficiency of homing as a function of local tissue properties is unclear (19). The risk of intravascular transplantation of cultured MSCs should also be taken into consideration (20,21). MSCs can be delivered by site-directed approaches in the treatment of impaired tissue (22). Since the submandibular glands of SD rats are substantially larger than those found in mice, and they can be targeted subcutaneously near the inferior border of the mandible, we elected to perform the transplantation of the hADSCs through an intraglandular route.

The SFR is one of the most visual indices for evaluating salivary gland function. A higher SFR was detected in the hADSC-injected group during the experimental period, as compared to the uninjected controls. However, a slight decline in the SFR in the hADSC-injected group was observed after 16 weeks, which may be attributed to a peak in the therapeutic effect. Although the salivary output of the submandibular gland in the hADSC treated group failed to recover competence to that of normal levels, an increase would be helpful for patients who suffer from dry mouth, and would alleviate symptoms and discomfort following xerostomia. The main reason for this is that the submandibular gland produces up to 90% of the salivary volume at rest and 40% under stimulation in humans (23).

ADSCs also have a multi-lineage differentiation potential (24). In a previous study, when hADSCs were co-cultured with murine salivary gland cells *in vitro*, only a low number of co-cultured hADSCs (13–18%) was observed to transdifferentiate into amylase-producing salivary gland epithelial cells, which represent acinar cells (25). In this study, ZsGreen-labeled hADSCs survived in the salivary glands of rats at 24 weeks post-irradiation, and a small proportion was observed as positive for the expression of NKCC-1, CK7 and CK14, which indicated transdifferentiation into acinar and ductal cells. Moreover, hADSCs produce multiple cytokines and chemokines, angiogenic and anti-apoptotic factors at bioactive levels (15). Additionally, saliva secretion is highly relevant to blood flow in the gland as the fluid component of saliva is derived from the local vascular bed in the gland (26). Our results showed enhanced angiogenesis and decreased apoptosis in the salivary glands of the hADSC-treated rats when compared to those of the untreated rats. In addition, both VEGF and HGF are factors considered to be important for the improvement of salivary glands. The expression of VEGF and HGF significantly increased 24 weeks post-irradiation, as shown by RT-qPCR. These results suggest that the paracrine effect of hADSCs participated in the protection against irradiation and improved the microstructure of rat submandibular glands.

In the present study, we injected hADSCs into the submandibular glands of SD rats. We found that the hADSCs survived in the salivary glands, and underwent differentiation into salivary epithelial cells in the xenogeneic environment. Surviving xenogeneic engrafted MSCs in fully immunocompetent adult recipients without immunosuppression have been suggested to be related to the immunoprivileged properties of MSCs (27). MSCs can avoid immunological rejection in humans and in animal models, and the mechanisms involved, while perplexing, remain poorly understood. Three broad mechanisms that may contribute to this effect are being considered and these include hypoimmunogenicity, the prevention of T cell responses and the induction of an immunosuppressive local microenvironment (28). Our results showing that long-term hADSC engraftment occurs in xenogeneic recipients without evidence of immunocompromise, and that hADSCs take part in tissue repair, are consistent with those of a previous study (22).

Overall, the present study demonstrates that the intraglandular transplantation of hADSCs can alleviate radiation-induced xerostomia, and our data may provide a reference suitable for subsequent clinical applications.

## Figures and Tables

**Table I:** Specific gene primers used for RT-qPCR.

Gene	Sense	Antisense	Annealing temperature (°C)	Product size (bp)
VEGF	5′-CAAACCTCACCAAAGCCAGC-3′	5′-ACGCGAGTCTGTGTTTTTGC-3′	59.97	187
HGF	5′-ACCCTGGTGTTTCACAAGCA-3′	5′-GCAAGAATTTGTGCCGGTGT-3′	59.97	182
COX-2	5′-GTGGAAAAGCCTCGTCCAGA-3′	5′-TCCTCCGAAGGTGCTAGGTT-3′	60.25	132
MMP-2	5′-CCCCATGTGTCTTCCCCTTC-3′	5′-TGGGCTGCCACAAGGAATAG-3′	60.03	169
GAPDH	5′-AATGCATCCTGCACCACCAA-3′	5′-GATGGCATGGACTGTGGTCA-3′	60.04	99
